# CD5 Immunoreactivity Is Associated With Longer Overall Survival in Thymic Carcinoma: A Brief Report

**DOI:** 10.1016/j.jtocrr.2025.100803

**Published:** 2025-02-11

**Authors:** Julia R. Naso, Sarah M. Jenkins, Julie A. Vrana, Justin W. Koepplin, Kenneth R. Olivier, Stephen D. Cassivi, Julian R. Molina, Anja C. Roden

**Affiliations:** a Department of Laboratory Medicine and Pathology, Mayo Clinic, Rochester, Minnesota; b Department of Pathology and Laboratory Medicine, Vancouver General Hospital, Vancouver, British Columbia, Canada; c Department of Pathology and Laboratory Medicine, University of British Columbia, Vancouver, British Columbia, Canada; d Department of Quantitative Health Sciences, Mayo Clinic, Rochester, Minnesota; e Department of Radiation Oncology, Mayo Clinic, Rochester, Minnesota; f Division of Thoracic Surgery, Mayo Clinic, Rochester, Minnesota; g Division of Medical Oncology, Mayo Clinic, Rochester, Minnesota

**Keywords:** Thymic carcinoma, CD5, Immunohistochemistry, Survival, Prognosis, Pathology

## Abstract

Thymic carcinomas are a heterogeneous group of potentially aggressive malignancies. We aimed to determine the prognostic significance of CD5, CD117, EZH2, POU2F3, BAP1, and MTAP immunohistochemical staining in thymic carcinomas. Immunohistochemistry was performed on 36 thymic carcinomas from patients with retrospectively collected survival data. Thirteen cases (36%) had CD5 staining in 50% or more tumor cells, considered positive staining. Positive CD5 immunohistochemical staining was significantly associated with longer overall survival (hazard ratio = 0.18, 95% confidence interval: 0.03–0.63, *p* = 0.005). Three-year overall survival was 48% (95% confidence interval: 27%–70%) for CD5 negative cases, and 100% for CD5 positive cases. Positive CD5 staining remained significantly associated with overall survival after adjusting for neoadjuvant treatment, M-stage, or incomplete resection (*p* = 0.01). The remaining immunohistochemical markers were not significantly associated with overall survival. Our study supports the notion that CD5 immunohistochemistry may have utility as a novel prognostic marker for thymic carcinoma.

## Introduction

Thymic carcinomas are malignant neoplasms with at least a subset of patients presenting at high stage and reported five-year overall survival rates ranging from 36 to 66%.^1^, ^2^, ^3^ Efforts to develop markers for the distinction of thymic carcinoma from thymoma have highlighted differences in immunoreactivity between individual thymic carcinomas. Possible associations between immunoreactivity and clinical outcomes are not well understood and are difficult to study given the rarity of thymic carcinoma.

CD5, CD117, EZH2, POU2F3, MTAP, and BAP1 have been proposed as useful markers for the distinction of thymic carcinoma from thymoma.^4^^,^^5^ None of these markers are 100% sensitive for thymic carcinoma,^4^^,^^5^ raising the question of whether heterogeneity in their expression among thymic carcinomas may be associated with differences in patient outcome. We explored the relationship between CD5, CD117, EZH2, POU2F3, MTAP, and BAP1 immunoreactivity and the overall survival (OS) and progression-free survival (PFS) of patients with thymic carcinoma, using a cohort with diverse histologic subtypes. Our results highlight a significant association between CD5 immunoreactivity and OS.

## Materials and Methods

This study was approved by the Mayo Clinic Institutional Review Board (10-003525, minimal risk study, consent was waived). Thymic carcinomas were classified according to the 2021 WHO classification and the eighth American Joint Committee on Cancer and Union for International Cancer Control staging system.^6^^,^^7^ Case identification and immunohistochemistry (IHC) was performed as described previously,^5^ with all patients undergoing surgery and receiving immediate postoperative care at Mayo Clinic. IHC used a Ventana BenchMark Ultra platform (Roche, Tuscon, AZ) and antibodies against CD5 (clone SP19, Cell Marque, Rocklin, CA), CD117 (YR145, Cell Marque), EZH2 (D2C9, Cell Signaling Technology, Danvers, MA), POU2F3 (polyclonal HPA019652, Atlas Antibodies, Bromma, Sweden), BAP1 (C-4, Santa Cruz Biotechnology, Dallas, TX), and MTAP (2G4, Abnova, Taipei, Taiwan). IHC was scored across the full slide or (for POU2F3 only) in a 200X hotspot. The percent of tumor cell staining required for a “positive” score was 50% or higher for CD5, 10% or higher for CD117, 80% or higher for EZH2, and 10% or higher for POU2F3, as previously identified.^4^^,^^5^ Survival times were calculated from the date of resection, through retrospective chart review.

Statistical analysis used SAS version 9.4 (SAS Institute Inc., Cary, NC) and RStudio (version 2022.07.1), and *p* values below 0.05 were considered statistically significant. Progression was defined as recurrence or new or progressive metastatic disease. Categorical variables were compared using Fisher’s exact tests. PFS and OS were assessed using likelihood ratio tests from Cox proportional hazards regression models. Median and three-year estimates for PFS and OS were calculated using the Kaplan-Meier method.

## Results

Thirty-six patients with thymic carcinoma who underwent tumor resection and had available follow-up data were included (Table 1). The immunohistochemical profiles of all patients were previously reported.^5^ IHC was performed on primary tumor resection specimens, except for one case with a complete response to neoadjuvant chemoradiation, for which IHC was performed on a recurrence specimen. The median age at resection was 57 years (range: 19–80 y), the median tumor size at resection was 5 cm (range: 0–14.5 cm), and the median follow-up time was 2.9 years (range: 0.2–17.8 y). Twelve patients received neoadjuvant therapy including neoadjuvant chemotherapy (n = 6), neoadjuvant chemoradiation (n = 4), or neoadjuvant radiation (n = 2). Neoadjuvant chemotherapy consisted of cisplatin with doxorubicin and cyclophosphamide (two to four cycles, n = 4), etoposide (two to four cycles, n = 4), carboplatin and paclitaxel with (six cycles), or without (four cycles) pembrolizumab (n = 1 each). Neoadjuvant radiation ranged from 20 to 61.2 cGy. Eight patients received both adjuvant and neoadjuvant treatment. Three-year OS and PFS were estimated at 64% (95% confidence interval [CI]: 47%–82%) and 45% (95% CI: 27%–63%), respectively. The median OS was 4.6 years and the median PFS was 2.6 years.

**Table 1:** Clinical and Pathologic Features and Outcomes of the Study Population (N = 36)

Clinical/Pathologic Feature^a^	All Carcinomas, n (%)	CD5 Negative, n (%)	CD5 Positive, n (%)	*p* Value for Association With CD5 Positivity^a^	*p* Value for OS	*p* Value for PFS
Total	36	23	13			
≥ 60 y old	16 (44)	7 (30)	9 (69)	0.038^b^	0.85	0.14
Male	20 (56)	10 (44)	10 (77)	0.083	0.41	0.73
Incomplete resection	8 (22)	6 (26)	2 (15)	0.68	0.14	0.56
Tumor size^c^ ≥ 5 cm	14 (54)	10 (63)	4 (40)	1.00	0.29	0.036^b^
pT stage 3 or 4	15 (42)	11 (48)	4 (31)	0.48	0.58	0.24
pN stage 1/2^d^	8 (40)	5 (42)	3 (38)	1.00	0.56	0.56
M-stage 1a/1b	6 (17)	6 (26)	0 (0)	0.068	0.20	0.17
Overall TNM stage^e^						
I	3 (15)	1 (9)	2 (22)	1.00^e^	0.77^e^	0.22^e^
II	3 (15)	1 (9)	2 (22)			
IIIA	3 (15)	2 (18)	1 (11)			
IIIB	1 (5)	1 (9)	0 (0)			
IVA	7 (35)	3 (27)	4 (44)			
IVB	3 (15)	3 (27)	0 (0)			
Carcinoma type						
Moderately differentiated squamous	4 (11)	4 (17)	0 (0)	0.075^f^	0.48^f^	0.22^f^
Poorly differentiated squamous	19 (53)	8 (35)	11 (85)			
Undifferentiated	3 (8)	1 (4)	2 (15)			
Small cell	2 (6)	2 (9)	0 (0)			
Adeno	2 (6)	2 (9)	0 (0)			
Mucoepidermoid	2 (6)	2 (9)	0 (0)			
Lymphoepithelial	2 (6)	2 (9)	0 (0)			
Adenosquamous	1 (3)	1 (4)	0 (0)			
Sarcomatoid	1 (3)	1 (4)	0 (0)			
Immunohistochemistry						
CD117 positive	31 (86)	18 (78)	13 (100)	0.14	0.23	0.48
EZH2 positive	29 (81)	17 (74)	12 (92)	0.38	0.62	0.37
POU2F3 positive	19 (53)	8 (35)	11 (85)	0.0058^b^	0.59	0.25
BAP1 loss	4 (11)	4 (17)	0 (0)	0.27	0.08	0.90
MTAP loss^g^	5 (14)	3 (14)	2 (15)	1.00	0.69	0.49
Additional therapies						
None	5 (14)	3 (13)	2 (15)	1.00	0.31	0.13
Adjuvant RT	25 (69)	14 (61)	11 (85)	0.26	0.31	0.37
Neoadjuvant RT	6 (17)	6 (26)	0 (0)	0.068	0.058	0.0013^b^
Adjuvant chemotherapy	14 (39)	8 (35)	6 (46)	0.72	0.26	0.13
Neoadjuvant chemotherapy	10 (28)	9 (39)	1 (8)	0.060	0.54	0.0014^b^
Any adjuvant treatment	27 (75)	16 (70)	11 (85)	0.44	0.31	0.84
Any neoadjuvant treatment	12 (33)	11 (48)	1 (8)	0.025^b^	0.22	0.0003^b^
Clinical outcome						
New or progressive metastatic disease or recurrence	20	15	5			
Died of disease	7	7	0			
Died of other cause	2	2	0			
Died of unknown cause	9	7	2			
Died of any cause	18	16	2			

OS, overall survival; PFS, progression-free survival; RT, radiation therapy.

a Fisher’s exact test for categorical variables.

b Statistically significant *p* values.

c Completely resected specimens only, including a size of 0 for pathologic complete response in neoadjuvantly treated tumor; denominator 26 (16 CD5 negative, 10 CD5 positive).

d N0 versus N1/2, excluding NX; Denominator 20 (12 CD5 negative, eight CD5 positive).

e For stages I to III versus IV, excluding cases that could not be assessed as no lymph nodes were sampled or there was the complete neoadjuvant response; denominator 20 (11 CD5 negative, nine CD5 positive).

f For squamous cell carcinoma versus other.

g Excluding one CD5 negative case for which MTAP status could not be determined owing to lack of internal positive control.

**Figure 1:**
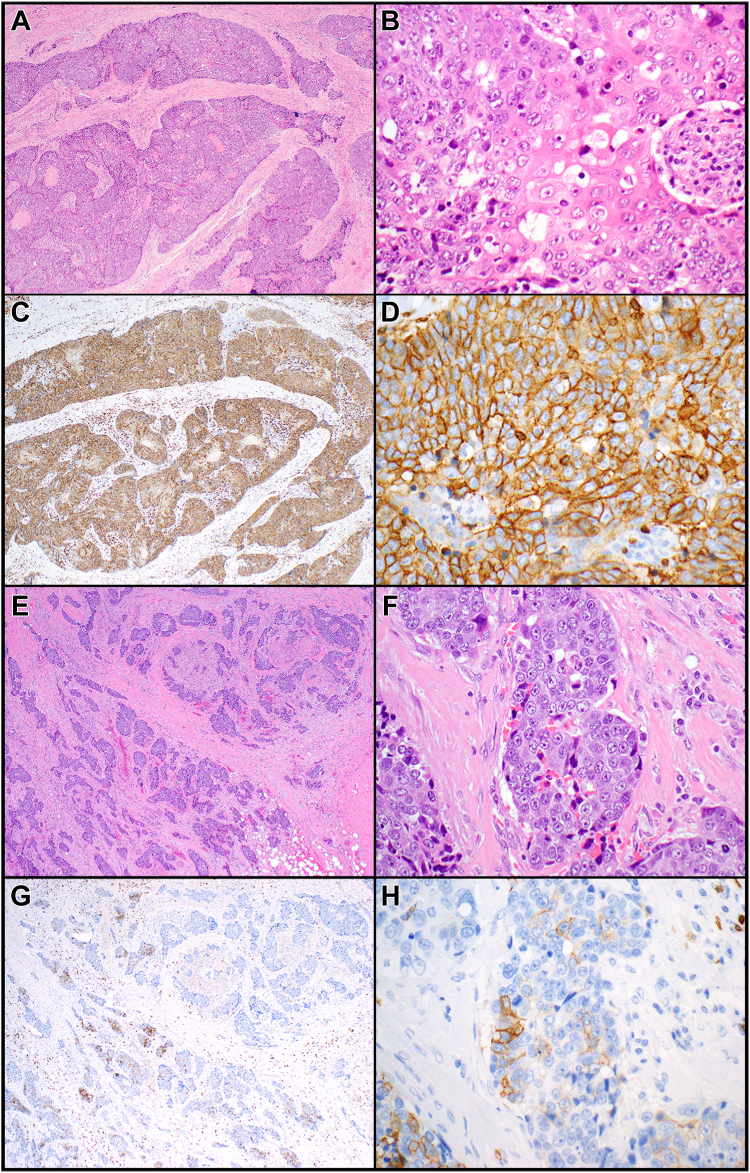
*(A–D)* Poorly differentiated thymic squamous cell carcinoma with more than 50% of tumor cells expressing CD5 (“CD5-positive”). *(A)* Variably sized nests of tumor cells are growing in a fibrotic stroma. *(B)* The tumor cells are polygonal and exhibit dyskeratotic features. *(C)* CD5 is expressed in essentially 100% of tumor cells and shows strong membranous staining *(D)*. (*E–H)* Poorly differentiated thymic squamous cell carcinoma with less than 50% of tumor cells expressing CD5 (“CD5-negative”). *(E)* Small nests of tumor cells are surrounded by a desmoplastic stroma. *(F)* The tumor cells are large and exhibit prominent nucleoli. *(G)* CD5 is only expressed in a few tumor cells (overall approximately 10% of tumor cell staining) showing weak to moderate membranous expression *(H)*. Magnification, H&E ×40 *(A, E)*, ×400 *(B, F)*, CD5 ×40 *(C, G)*, ×400 *(D, H)*. H&E, hematoxylin and eosin.

**Figure 2:**
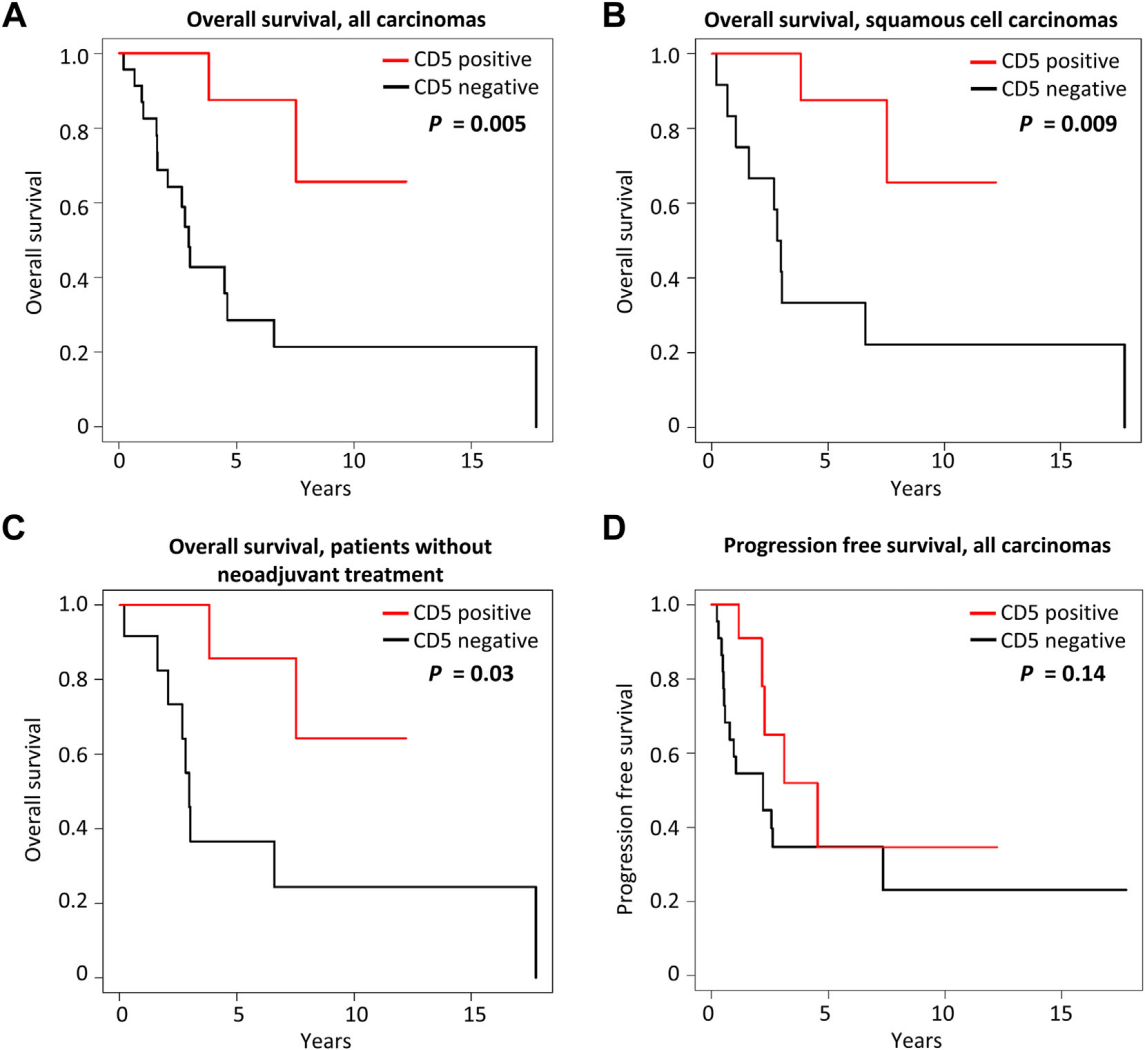
Kaplan-Meier curves for the association of positive CD5 staining with *(A)* overall survival of all thymic carcinomas, *(B)* overall survival of thymic squamous cell carcinomas, *(C)* overall survival of patients without neoadjuvant treatment, and *(D)* progression-free survival of all thymic carcinomas. *p* values from likelihood ratio tests are shown.

CD5 immunoreactivity (Fig. 1*A–H*) was significantly associated with longer OS (hazard ratio [HR] = 0.18, 95% CI: 0.03–0.63, *p* = 0.005; Fig. 2*A* and Table 1). CD5 negative cases had a three-year OS of 48% (95% CI: 27%–70%) whereas CD5 positive cases had a three-year OS of 100%. The median OS was 3.0 years (95% CI: 1.6–6.6 y) for CD5 negative cases and could not be estimated for CD5 positive cases owing to a lack of events. All seven patients with disease-specific mortality had CD5-negative carcinomas. When analyzing only thymic squamous cell carcinomas (n = 23), positive CD5 staining remained significantly associated with OS (HR = 0.17, 95% CI: 0.03–0.65, *p* = 0.009; Fig. 2*B*). CD5 also remained significantly associated with OS when including all cases except small cell carcinomas and mucoepidermoid carcinomas (i.e., only non-neuroendocrine, non–salivary gland-type tumors included; HR = 0.15, 95% CI: 0.02–0.56, *p* = 0.003). Other clinicopathologic variables (Table 1) were not significantly associated with OS.

Positive CD5 staining was also significantly associated with the patient’s age of 60 years and above (*p* = 0.038), positive POU2F3 staining (*p* = 0.0058), and absence of neoadjuvant treatment (*p* = 0.025), but not other clinicopathologic factors evaluated (Table 1). Most CD5-positive cases were squamous cell carcinomas, though this association did not reach statistical significance (*p* = 0.075). Among patients who did not receive neoadjuvant treatment (n = 24), positive CD5 staining was still significantly associated with OS (HR = 0.21, 95% CI: 0.03–0.85, *p* = 0.03; Fig. 2*C*).

Bivariate analysis was performed for CD5 in combination with the clinical variables that had the largest effects in the univariate analysis. Positive CD5 staining remained significantly associated with OS after adjusting for either neoadjuvant radiation (HR = 0.21, 95% CI: 0.03–0.79, *p* = 0.02), neoadjuvant chemotherapy or radiation (HR = 0.19, 95% CI: 0.03–0.71, *p* = 0.01), incomplete resection (HR = 0.20, 95% CI: 0.03–0.73, *p* = 0.01), or M1 stage (HR = 0.19, 95% CI: 0.03–0.71, *p* = 0.01). In addition, CD5 remained significantly associated with OS after adjusting for age (HR = 0.15, 95% CI: 0.02–0.57, *p* = 0.01).

CD5 negative cases had a three-year PFS of 35% (95% CI: 14%–55%), whereas CD5 positive cases had a three-year PFS of 65% (95% CI: 32%–98%). This difference was not statistically significant (HR = 0.48, 95% CI: 0.16–1.26, *p* = 0.14; Fig. 2*D* and Table 1). Shorter PFS was significantly associated with tumor size of 5 cm and larger (HR = 3.0, 95% CI: 1.1–9.8, *p* = 0.036) and neoadjuvant treatment (radiation: HR = 6.7, 95% CI: 2.3–17.9, *p* = 0.0013; chemotherapy: HR = 4.52, 95% CI: 1.8–11.2, *p* = 0.0014; radiation or chemotherapy: HR = 5.3 95% CI: 2.2–13.6, *p* = 0.0003), but not with other variables (Table 1).

## Discussion

Associations between clinical outcomes and immunoreactivity for markers of thymic carcinoma are not well understood. We report that CD5 immunoreactivity is significantly associated with the OS of patients with thymic carcinoma. This association remained significant when evaluating only patients with thymic squamous cell carcinomas, patients with non-neuroendocrine and non–salivary gland–type tumors, or patients who only underwent resection without neoadjuvant therapy. Positive CD5 staining was also significantly associated with the ages of 60 years and above and management without neoadjuvant treatment.

CD5 is a cysteine-rich scavenger receptor family glycoprotein constitutively expressed as a co-receptor on T-cells and a subset of B-cells.^8^ CD5 plays a role in immune tolerance and lymphocyte selection, with thymocytes of CD5-deficient mice showing hyper-responsiveness to T-cell receptor activation.^9^ Regulation of CD5 expression on T-cells has been suggested as one mechanism through which malignancies may evade immune response.^10^ CD5 expression on carcinoma cells may therefore affect the capacity of carcinoma cells to evade immune responses, but this hypothesis remains to be tested. Our study highlights the clinical relevance of further investigation of possible mechanisms through which CD5 expression on epithelial cells may influence tumor behavior. The only prior report of an association between CD5 staining and thymic carcinoma outcomes found that positive CD5 staining was associated with longer PFS but did not reach significance for association with OS (n = 27).^11^ Conversely, our study reached statistical significance for the association of CD5 with OS but not PFS. This and the previous study together support an association between CD5 staining and favorable outcomes. The previous study was focused on metastatic or unresectable cases,^11^ whereas 30 out of 36 cases in our study were negative for distant metastatic disease, supporting generalizability to non-metastatic cases. Furthermore, it is unclear which criteria for CD5 expression in tumor cells and which CD5 antibody clone was used in the prior study.

Overall, 36% of thymic carcinomas in our study were positive for CD5 (i.e., ≥50% of tumor cell staining). Previous studies have reported that 39% to 70% of thymic carcinomas are CD5 positive,^1^^,^^12^, ^13^, ^14^ with thresholds ranging from any degree of staining to strong or intermediate intensity staining in over 10% of tumor cells. The relatively low proportion of CD5-positive cases in our study is likely related to a high threshold for positivity, which was selected to allow 100% specificity for the distinction of thymic carcinoma versus thymoma.^4^^,^^5^

In our study, positive CD5 staining was significantly associated with POU2F3 staining.^4^^,^^5^ A correlation between CD5 and POU2F3 immunoreactivity has previously been noted,^15^^,^^16^ but the biological relationship of these markers remains uncertain. POU2F3 is a regulator of tuft cell differentiation expressed in multiple malignancies including thymic squamous cell carcinoma and a subset of small cell carcinoma.^15^^,^^17^^,^^18^ One previous study reported a tendency for thymic carcinomas with CD117 immunoreactivity to have shorter OS, but this association did not reach statistical significance (*p* = 0.05, n = 18),^19^ and no association between CD117 and survival was evident in our study.

The median OS of patients in our study (4.6 y) was similar to that in prior studies of thymic carcinoma (e.g., 4.0 y^20^ and 6.6 y^21^). Recognized independent prognostic factors for thymic carcinoma include stage, complete resection, and adjuvant treatment,^21^ but these factors were not significantly associated with OS or PFS in our study, possibly at least in part owing to the limited number of patients included in our study. Tumor size has been associated with disease-free survival^2^ and was significantly associated with PFS in our study.

Limitations of our study include its retrospective nature. The limited number of patients and events in survival analysis precluded the use of more complex multivariate models and the modest sample size limited statistical power for detecting small differences in survival. We also caution that differences in how CD5 IHC is scored and potential differences in the performance of various CD5 antibody clones may affect the generalizability of our results to archival CD5 data from other laboratories.

Overall, we find that CD5 immunoreactivity in thymic carcinoma is associated with longer OS. Our study underscores the potential clinical relevance of variability in CD5 staining as a prognostic marker for thymic carcinoma, and the need for further investigation of mechanisms by which CD5 expression may impact the survival of patients with thymic carcinoma.

## CRediT Authorship Contribution Statement

**Julia R. Naso:** Data acquisition, Data analysis, Writing - original draft, Writing - review & editing, Final approval.

**Sarah M. Jenkins:** Statistical analysis, Final approval.

**Julie A. Vrana:** Data acquisition, Final approval.

**Justin W. Koepplin:** Data acquisition, Final approval.

**Kenneth R. Olivier:** Writing - review & editing, Final approval.

**Stephen D. Cassivi:** Writing - review & editing, Final approval.

**Julian R. Molina:** Writing - review & editing, Final approval.

**Anja C. Roden:** Conceptualization, Funding acquisition, Data acquisition, Writing - review & editing, Final approval.

## Disclosure

Dr. Roden reports honoraria from the Pathology Learning Center for educational lectures; royalties from Up-To-Date for educational material; position on the advisory board of Sanofi Oncology and AstraZeneca; and is a consultant to Bristol Myers Squibb. The remaining authors declare no conflict of interest.
